# The art of obtaining a high yield of cell-free DNA from urine

**DOI:** 10.1371/journal.pone.0231058

**Published:** 2020-04-06

**Authors:** Elien Augustus, Kaat Van Casteren, Laure Sorber, Peter van Dam, Geert Roeyen, Marc Peeters, Alex Vorsters, An Wouters, Jo Raskin, Christian Rolfo, Karen Zwaenepoel, Patrick Pauwels

**Affiliations:** 1 Center for Oncological Research Antwerp (CORE), University of Antwerp (UA), Wilrijk, Belgium; 2 Laboratory of Pathological Anatomy, Antwerp University Hospital (UZA), Edegem, Belgium; 3 Biomedical Quality Assurance Research Unit, Department of Public Health and Primary Care, KU Leuven (KUL), Leuven, Belgium; 4 Multidisciplinary Breast Unit, Antwerp University Hospital (UZA), Edegem, Belgium; 5 Department of Hepato-Pancreato-Biliary and Transplant Surgery, Antwerp University Hospital (UZA), Edegem, Belgium; 6 Department of Oncology, Antwerp University Hospital, Antwerp (UZA), Belgium; 7 Centre for the Evaluation of Vaccination, Vaccine & Infectious Disease Institute, University of Antwerp (UA), Wilrijk, Belgium; 8 Department of Pulmonology and Thoracic Oncology, Antwerp University Hospital (UZA), Edegem, Belgium; 9 Thoracic Medical Oncology and the Early Clinical Trials at the University of Maryland Marlene and Stewart Greenebaum Comprehensive Cancer Center (UMGCCC), Baltimore, Maryland, United States of America; Abel Salazar Institute of Biochemical Sciences, University of Porto/Portuguese Oncology Institute of Porto, PORTUGAL

## Abstract

Although liquid biopsies offer many advantages over tissue biopsies, they are not yet standard practice. An important reason for the lack of implementation is the unavailability of well standardized techniques and guidelines, especially for pre-analytical conditions which are an important factor causing the current sensitivity issues. To overcome these limitations, we investigated the effect of several pre-analytical conditions on the concentration of cell-free DNA (cfDNA) and cellular genomic DNA (gDNA) contamination. Urine samples from healthy volunteers (HVs) and cancer patients were collected and processed according to specific pre-analytical conditions. Our results show that in samples with a relatively small volume more than 50% of the cfDNA can be found in the first 50 mL of the urine sample. The total DNA concentration increased again when samples were collected more than 3.5 hours apart. Adding preservative to urine samples is recommended to obtain high concentrations of cfDNA. To remove the cellular content, high speed centrifugation protocols as 4,000g 10min or 3,000g 15min are ideal for urine collected in cfDNA Urine Preserve (Streck). Although this study was a pilot study and needs to be confirmed in a larger study population, clear trends in the effect of several pre-analytical conditions were observed.

## Introduction

Currently, molecular tumor profiling largely depends on the availability of a tissue biopsy for solid cancer types [1–3]. Even though tissue biopsies are sometimes required at several time points during the patients’ disease course, obtaining a tissue biopsy can be problematic as this is often highly invasive and involves a certain risk for the patient depending on the location of the tumor. Furthermore, the biopsy does not always contain a sufficient amount of tumor cells for molecular profiling. In addition, since a tissue biopsy only represents a snapshot of the tumor, studying tumor heterogeneity is often not feasible with tissue biopsies [4–6].

An alternative method to characterize the tumor on a molecular level is liquid biopsy. It enables the analysis of cancer associated biomarkers in liquid biological material, typically blood. A liquid biopsy consists of several circulating components derived from non-malignant tissue as well as from cancer tissue. These circulating components include circulating cell-free DNA (cfDNA), circulating cell-free RNA (cfRNA), microRNA (miRNA), tumor proteins, circulating tumor cells (CTCs), extracellular vesicles (EVs) and tumor educated platelets (TEPs) [4, 7, 8]. Liquid biopsies have great potential to overcome the limitations associated with tissue biopsies. In particular, they enable repeated sampling of cancer patients to facilitate a more detailed patient monitoring, provide a more accurate and relevant representation of the current disease status, allow the investigation of tumor heterogeneity and can provide additional information for treatment selection [6, 9]. In lung cancer, the use of the Cobas *Epidermal Growth Factor Receptor (EGFR)* Mutation Test v2 (Roche, Basel Switzerland) is approved for the detection of *EGFR* variants by cfDNA analysis by the Food and Drug Administration (FDA) for treatment selection [10–12]. In May 2019 the therascreen® *PIK3CA* RGQ PCR Kit received U.S. regulatory approval as a companion diagnostic to help identifying breast cancer patients eligible for treatment with PIQRAY (Alpelisib). Currently, liquid biopsy is mainly used in specific clinical situations, for example when tissue availability is limited and/or insufficient for molecular testing [2, 13].

Other fluids like urine, saliva and cerebrospinal fluid can also be used, but are less researched compared to blood samples. In general, cfDNA that is released in the blood circulation by apoptosis or necrosis is between 116–161 bp long [14–16]. Data of circulating fetal DNA in urine suggest that urinary cfDNA is even more fragmented, ranging from 40–250 bp [17–19]. Recently the focus is being shifted towards the research of urine for molecular tumor profiling. Compared to blood sampling, urine-based biopsies are completely noninvasive, enable self-sampling at home and consequently increase patient comfort for single collections or repeated sampling. Therefore, they offer a promising alternative to tissue and blood-based samples [20, 21]. Despite the high potential of urine as a liquid biopsy, no standardized protocol for pre-analytical handling of urine to preserve cfDNA for downstream applications are available, which is problematic because 32–75% of all testing errors are the result of issues that arise during the pre-analytical phase [22–24]. The use of appropriate pre-analytical conditions is essential to achieve a high yield of cfDNA and to reach an accurate and sensitive detection of molecular biomarkers in urine. The optimization and standardization of the pre-analytical conditions in blood already have been studied by many research groups [24–29]. In contrast, only a few studies have been published in urine [21, 23, 30, 31]. For this reason, we investigated several pre-analytical conditions to determine those most appropriate to generate high quality urine samples suitable for molecular analysis. Ideally, the presence of long genomic DNA (gDNA) fragments (>500 bp), derived from intact non-malignant cells from the urinary tract, should not dilute the already low concentration of tumor-derived cfDNA in the samples. This dilution will inevitably decrease the sensitivity of the used detection techniques and make accurate detection of molecular biomarkers more difficult. To increase sensitivity of urine analysis, the aim of this study was to study the influence of different pre-analytical conditions on the concentration of cfDNA and genomic DNA.

## Materials & methods

### Sample collection

This study was approved by the Ethical Committee of the University Hospital of Antwerp (UZA) (B300201422715). To study the different pre analytical conditions, urine samples were obtained from 39 healthy volunteers (HVs) and 14 cancer patients (colorectal cancer (CRC), pancreatic ductal adenocarcinoma (PDAC), breast cancer and non-small cell lung cancer (NSCLC)) with metastases (stage IV), after signing informed consents (Table 1). The cancer patients and HVs had to be at least 18 years old. Sample collection and patient inclusions took place in the UZA from December 2017 until February 2019. The study was explained to the patients and the informed consents were taken by the study coordinator of the Oncology Unit and the nurses of the Multidisciplinary Breast Unit of the UZA. Samples were collected using sterile 125 mL polypropylene containers (Deltalab, Barcelona, Spain) between 8 AM and 6 PM. All samples were processed immediately, unless specified otherwise. Unfortunately, the total DNA concentration of some collected samples was too low (<10 ng/mL) and were excluded as described below. We evaluated different parameters in this study, therefore the sample size per parameter was too small to generate significant results.

**Table 1:** Characteristics from all HVs and cancer patients included in this study.

**1. First-void versus random urine collection**					
**HVs (n = 5)**			**Patients (n = 2)**		
**Age, y**			**Age, y**		
** **	Average	29	** **	Average	66
** **	Range	24–37	** **	Range	54–73
**Sex, n (%)**			**Sex, n (%)**		
** **	Female	5 (100)	** **	Female	2(100)
** **	Male	0 (0)	** **	Male	0 (0)
**2. Collection time points**					
**HVs (n = 4)**			**Patients (n = 0)**		
**Age, y**			**Age, y**		
** **	Average	27	** **	Average	X
** **	Range	23–30	** **	Range	X
**Sex, n (%)**			**Sex, n (%)**		
** **	Female	2 (50)	** **	Female	X
** **	Male	2 (50)	** **	Male	X
**3. Effect of preservatives**					
**HVs (n = 5)**			**Patients (n = 3)**		
**Age, y**			**Age, y**		
** **	Average	26	** **	Average	65
** **	Range	22–31	** **	Range	59–71
**Sex, n (%)**			**Sex, n (%)**		
** **	Female	3 (60)	** **	Female	2 (67)
** **	Male	2 (40)	** **	Male	1 (33)
**4. Effect of storage temperature and time**					
**FRESH URINE**					
**HVs (n = 4)**			**Patients (n = 2)**		
**Age, y**			**Age, y**		
** **	Average	25	** **	Average	51
** **	Range	22–27	** **	Range	35–66
**Sex, n (%)**			**Sex, n (%)**		
** **	Female	4 (100)	** **	Female	1 (50)
** **	Male	0 (0)	** **	Male	1 (50)
**PRESERVATIVE**					
**HVs (n = 4)**			**Patients (n = 0)**		
**Age, y**			**Age, y**		
** **	Average	26	** **	Average	X
** **	Range	23–28	** **	Range	X
**Sex, n (%)**			**Sex, n (%)**		
** **	Female	3 (75)	** **	Female	X
** **	Male	1 (25)	** **	Male	X
**5. Preparation of cell-free urine by centrifugation**					
**FRESH URINE**					
**HVs (n = 3)**			**Patients (n = 0)**		
**Age, y**			**Age, y**		
** **	Average	26	** **	Average	X
** **	Range	24–28	** **	Range	X
**Sex, n (%)**			**Sex, n (%)**		
** **	Female	3 (100)	** **	Female	X
** **	Male	0 (0)	** **	Male	X
**PRESERVATIVE**					
**HVs (n = 4)**			**Patients (n = 4)**		
**Age, y**			**Age, y**		
** **	Average	27	** **	Average	66
** **	Range	24–29	** **	Range	51–79
**Sex, n (%)**			**Sex, n (%)**		
** **	Female	4 (100)	** **	Female	4 (100)
** **	Male	0 (0)	** **	Male	0 (0)

HV = healthy volunteers, y = years.

### First-void versus random urine collection

**Fig 1:**
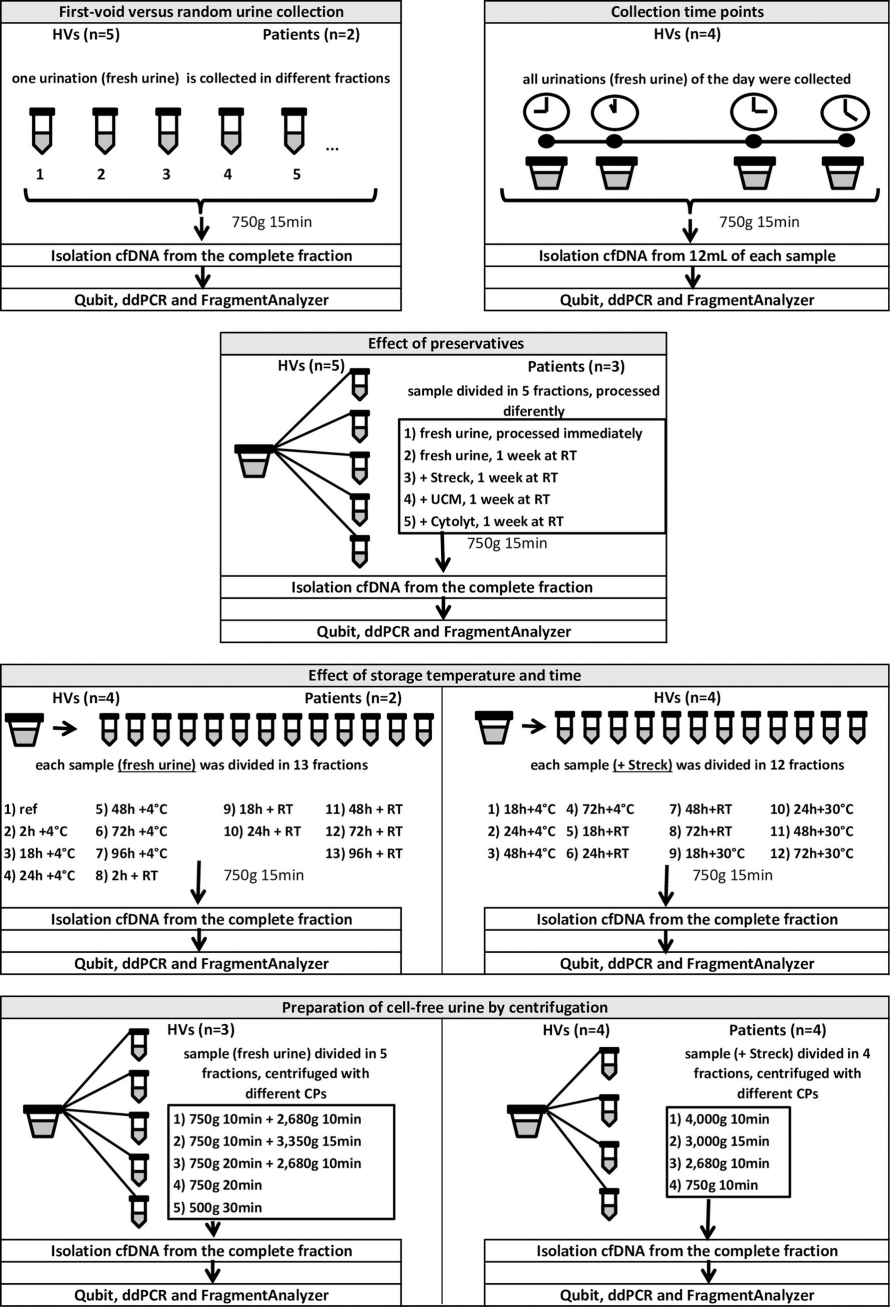
Flowchart of all the investigated pre-analytical conditions. Each condition is shown in a different frame. The number of HVs and patients from which samples were collected and analyzed are displayed on top of each frame. Below the experimental set-up and the used techniques are shown. CP = centrifugation protocol, HVs = healthy volunteers, RT = room temperature, Streck = cfDNA Urine Preserve, UCM = Urine conservation medium.

Fresh urine (= samples without preservative) from nine HVs (three male and six female) was collected in consecutive fractions of 20–30 mL. For two metastatic cancer patients (two female), urine was collected in two separate containers, as it was unfeasible to collect various consecutive fractions of 20–30 mL. P1 collected one urination in two fractions of 32.5 mL (first fraction) and 48 mL (second fraction). P2 collected two fractions of 55 mL (first fraction) and 90 mL (second fraction). For HV1, 12mL of urine per fraction of 20–30 mL was used for isolation. However the measured concentration of cfDNA was very low in some of the urine fractions. Therefore we isolated the cfDNA from the complete urine fractions of all other HVs and cancer patients. The urine of the four HVs and patient 1 (P1) was collected without preservative. The urine of patient 2 (P2) however was supplemented with preservative (cfDNA Urine Preserve, Streck Inc, La Vista, Nebraska, United States of America) and centrifuged according to the manufacturer’s recommendations. In four of the HVs samples (three male and one female), the total DNA concentration was too low (<10 ng/mL), therefore these samples were not analyzed (Fig 1).

### Collection time points

Again, five HVs (three male and two female) who agreed to collect multiple samples during one day, including the first collection of the day, were recruited. Each HV provided at least three individual samples, with a maximum of five samples which were not collected at fixed timings. Total volumes of the samples varied between 55 mL and 470 mL. For one HV, the first collection of the day was missed. All samples were gently mixed by three-time inversion immediately before cfDNA isolation. The DNA concentration in all samples from one HV (male) was very low (<10 ng/mL) and hence the data from this HV was not taken into consideration. Fractions of 12 mL were separated from the total sample and processed individually (Fig 1).

### Effect of preservatives

The stabilizing effect of three different preservatives was investigated: i) cfDNA Urine Preserve (Streck Inc), a commercially available preservative specifically for urinary cfDNA, ii) Urine conservation medium (UCM, University of Antwerp, Wilrijk, Belgium), an in-house developed preservative, designed to preserve human papillomavirus (HPV) DNA and total human DNA in urine samples [32] and iii) ThinPrep Cytolyt Solution (Hologic Inc, Marlborough, Massachusetts, United Stated of America), a preservative for cytological specimens.

Each sample, derived from one of the five HVs (two male and three female), was divided into five separate fractions after sample homogenization. Three fractions were supplemented with the preservatives described previously. The whole fractions were isolated. These fractions were stored at room temperature (RT) for one week. One fraction without preservative was also stored at RT for one week. The other fraction without preservative was processed immediately (<2h) after collection and served as a reference. Urine samples from three cancer patients (one male and two female) were also included in this study. Due to the limited volume of the patient samples (60–75 mL) and the volume requirements for the preservatives (Streck preservative requires ≥25 mL urine), not all testing conditions could be evaluated (Tables 2 and 3).

**Table 2:** Division of three patient urine samples into separate fractions to which preservative was added or not.

Volume (mL)	Fractions
75	No preservative
	UCM
	Streck
70	No preservative
	UCM
	Streck
60	Reference (processed immediately)
	No preservative
	UCM
	Cytolyt

Streck = cfDNA Urine Preserve, UCM = Urine conservation medium.

**Table 3:** Available testing combinations using two patient samples.

Storage temperature	Storage time (hours)	P1 volume: 20 mL	P2 volume: 66 mL
RT	0	Yes	Yes
	2	N/a	N/a
	18	N/a	Yes
	24	N/a	Yes
	48	N/a	Yes
	72	N/a	Yes
	96	N/a	Yes
4°C	2	N/a	N/a
	18	N/a	Yes
	24	Yes	Yes
	48	Yes	Yes
	72	Yes	Yes
	96	N/a	Yes

N/a = Not analyzed, P1/2 = patient 1/2, RT = Room temperature.

### Effect of storage temperature and time

Fresh urine samples were collected from five HVs (one male and four female) and three patients (one male and two female), of which one male HV sample and one female patient sample had very low concentration of total DNA (<10 ng/mL). These were not taken into account during analysis. The remaining samples were divided into 13 fractions of 12 mL after homogenization. The complete fractions were isolated. One fraction was used as a reference and processed immediately after collection. The other 12 fractions were stored for different periods of time (2, 18, 24, 48, 72 and 96 hours after collection) at RT or 4°C. Due to volume limitations, not all testing combinations could be evaluated in one female HV and two patient samples (Table 3).

In addition, cfDNA Urine Preserve (Streck) was added to urine samples from five HVs (two male and three female), of which one male HV had a low DNA concentration (<10 ng/mL) and was not analyzed further. Each sample was divided into 12 fractions whenever possible (Table 4). These fractions were stored for different periods of time (18, 24, 48 and 72 hours) at varying temperatures (4°C, RT, 30°C). Due to limitations in sample volume, not all of these testing combinations could be evaluated for every HV (Table 4).

**Table 4:** Available testing combinations based on sample volume for four samples.

Storage temperature	Storage time (hours)	HV1 volume: 170 mL	HV2 volume: 30 mL	HV3 volume: 170 mL	HV4 volume: 80 mL
4°C	18	Yes	N/a	Yes	N/a
	24	Yes	N/a	Yes	N/a
	48	Yes	N/a	Yes	N/a
	72	Yes	N/a	Yes	N/a
RT ~22°C	18	Yes	N/a	Yes	Yes
	24	Yes	Yes	Yes	Yes
	48	Yes	Yes	Yes	Yes
	72	Yes	N/a	Yes	Yes
30°C	18	Yes	N/a	Yes	Yes
	24	Yes	Yes	Yes	Yes
	48	Yes	Yes	Yes	Yes
	72	Yes	N/a	Yes	Yes

HV = healthy volunteer, N/a = Not analyzed, RT = Room temperature, Streck = cfDNA Urine Preserve, UCM = Urine conservation medium.

### Preparation of cell-free urine by centrifugation

After homogenization, fresh urine samples from five HVs (one male and four female) were each divided into five fractions of equal volume. Per fraction a different centrifugation protocol was used (Table 5). The total DNA concentration of two urine samples (one male and one female) was too low (<10 ng/mL) to generate reliable results, therefore these samples were not analyzed. Concurrently, cfDNA Urine Preserve (Streck) was added to urine samples from five HVs (one male and four female) and six metastatic cancer patients (one male and five female). After homogenization, each sample was divided in four fractions of equal volume and centrifuged according to a different protocol (Table 5). The whole fractions were isolated, similar to the experiment in which centrifugation protocols were studied in fresh urine samples. Unfortunately, the total DNA concentration of three urine samples (from one male HV, one male patient and one female patient) was very low (<10 ng/mL), as a consequence, those samples were not taken into account when analyzing our data (Fig 1).

**Table 5:** Centrifugation protocols used in the experimental setup for fresh urine and urine with a preservative.

**Fresh urine**		
**CP 1**	**750g 10min + 2,680g 10min**	*In-house* centrifugation step that is used in cell culture laboratory to remove cells in samples followed by the centrifugation step recommended by Streck for urine samples with preservative
**CP 2**	**750g 10min + 3,350g 15min**	*In-house* centrifugation step (see CP1) followed by the maximum centrifugation speed of the used centrifuge
**CP 3**	**750g 20min + 2,680g 10min**	CP1, but adjusted to 20min to study the effect of the centrifugation time on gDNA contamination
**CP 4**	**750g 20min**	CP3, without the second centrifugation step to study the effect/need of a second centrifugation step
**CP 5**	**500g 30min**	CP4, but with increased duration and lower speed to study the effect on gDNA contamination
**Urine with preservative**		
**CP I**	**4,000g 10min**	Centrifugation protocol based on literature: Brisuda et al., Urinary Cell-Free DNA Quantification as Non-Invasive Biomarker in Patients with Bladder Cancer, 2015 [38]
**CP II**	**3,000g 15min**	Centrifugation step recommended by ZymoResearch (Irvine, California, USA)
**CP III**	**2,680g 10min**	Centrifugation step recommended by Streck for urine samples with preservative
**CP IV**	**750g 10min**	In-house centrifugation step that is used in cell culture laboratory to remove cells in samples

CP = centrifugation protocol, gDNA = genomic DNA, min = minutes.

### cfDNA analysis

To remove the cellular content, all fresh urine samples were centrifuged at 750g for 15 minutes using a swing bucket centrifuge (Eppendorf AG, Hamburg, Germany) unless specified otherwise. cfDNA isolation was performed using the Quick-DNA Urine Kit (ZymoResearch, Irvine, California, USA) according to the manufacturer’s protocol and cfDNA was eluted in a volume of 50–80 μL. After isolation, the samples were stored at -20°C until further analysis. All urine samples, were analyzed using three techniques assessing either the total DNA concentration or the length of the DNA fragments (cfDNA and gDNA). Qubit DNA analysis was performed using the Qubit dsDNA High Sensitivity Assay Kit on the Qubit 3.0 Fluorometer (ThermoFisher Scientific, Walham, Massachusetts, USA) according to the manufacturer’s protocol. All samples were tested in triplicate using 2 μL.

Digital droplet PCR (ddPCR) analysis was performed using the QX200 ddPCR equipment (Bio-Rad, Hercules, California, USA). Following assays were used according to the manufacturer’s instructions: the ddPCR *KRAS* G12/G13 Screening Multiplex Kit (Bio-Rad Laboratories), PrimePCR ddPCR Mutation Assay Kit consisting of *PIK3CA* WT, p.E542K (Inc.#1863131), p.E545K (Inc. #1863132) and p.H1047R (Inc. #1863133) (Bio-Rad Laboratories). A master mix was made, consisting of 10.5 μL 2x digital PCR Supermix for Probes—No dUTP (Bio-Rad, #1863024) and 1 μL of the primers and probes mix. DNA template (8–9 μL) was added, PCR amplification was performed on the Veriti Thermal Cycler (ThermoFisher Scientific). The *KRAS* or *PIK3CA* mutation status of each sample was determined using the QX200 digital droplet reader (Bio-Rad Laboratories) and the Quantasoft Software v1.7 (Bio-Rad Laboratories).

To study the length of the DNA fragments, the DNF-474-33 –High Sensitivity NGS Fragment Analysis kit for the FragmentAnalyzer (Applied Biosystems, Foster City, California, USA) was used according to the manufacturer’s protocol. This kit has was chosen for its high sensitivity in the short fragment region but unfortunately it has an upper limit of only 6000 base pairs (bp), which was insufficient for some of the samples. Each sample was tested using 2 μL. Data analysis was performed using the PROSize 3.0 software. An additional ‘smear analysis’ was performed on the data after the run. In this ‘smear analysis’ the relative amount of DNA present between following boundaries was calculated: 40–250 bp and 500–5500 bp. Short fragmented cfDNA was represented by the region 40–250 bp and 500–5500 bp represents the long fragmented gDNA. The boundary of 40 bp and 250 bp was based on literature, since studies show that cfDNA fragments are smaller than 250 bp and potentially even as small as 40 bp in urine [17–19]. The standard deviation of the samples analyzed with the FragmentAnalyzer was calculated by testing a variation of samples with low and high concentrations of DNA (the tested samples have a coefficient of variation (CV) below 15%).

## Results

### First-void versus random urine collection

**Fig 2:**
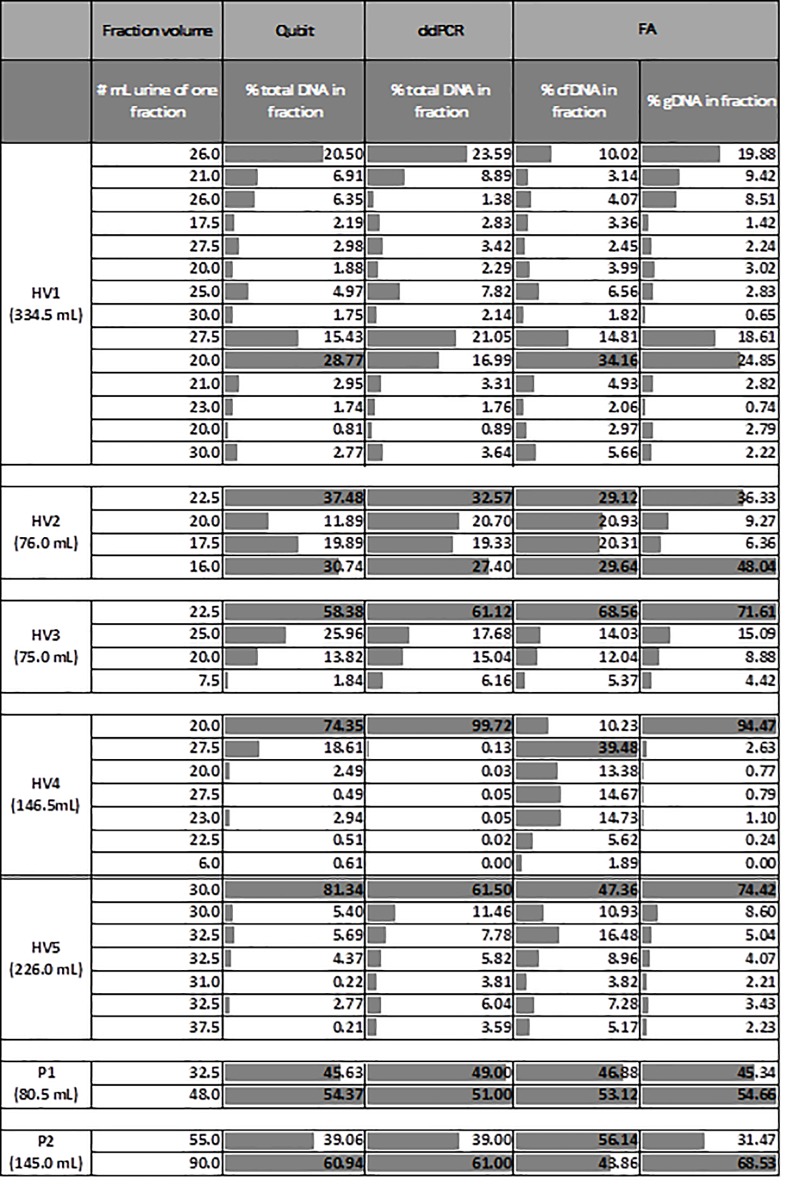
The percentage of the total DNA, cfDNA and gDNA present in the different fractions of one urine sample derived from five HVs and two patients. The concentration of total DNA was measured by Qubit Fluorometer and ddPCR. The samples were also analyzed with the FragmentAnalyzer. The percentage of cfDNA and gDNA of each fraction within the entire sample is also visually represented by the grey bars. 100% cfDNA = total amount cfDNA present in the sample according to the measurements of our research team with the mentioned technique. X% cfDNA = X% cfDNA of the 100% cfDNA that is present in the sample based on our measurements. cfDNA = circulating cell-free DNA, ddPCR = digital droplet PCR, FA = FragmentAnalyzer, gDNA = genomic DNA, HV = healthy volunteer, P1/2 = patient 1/2.

**Fig 3:**
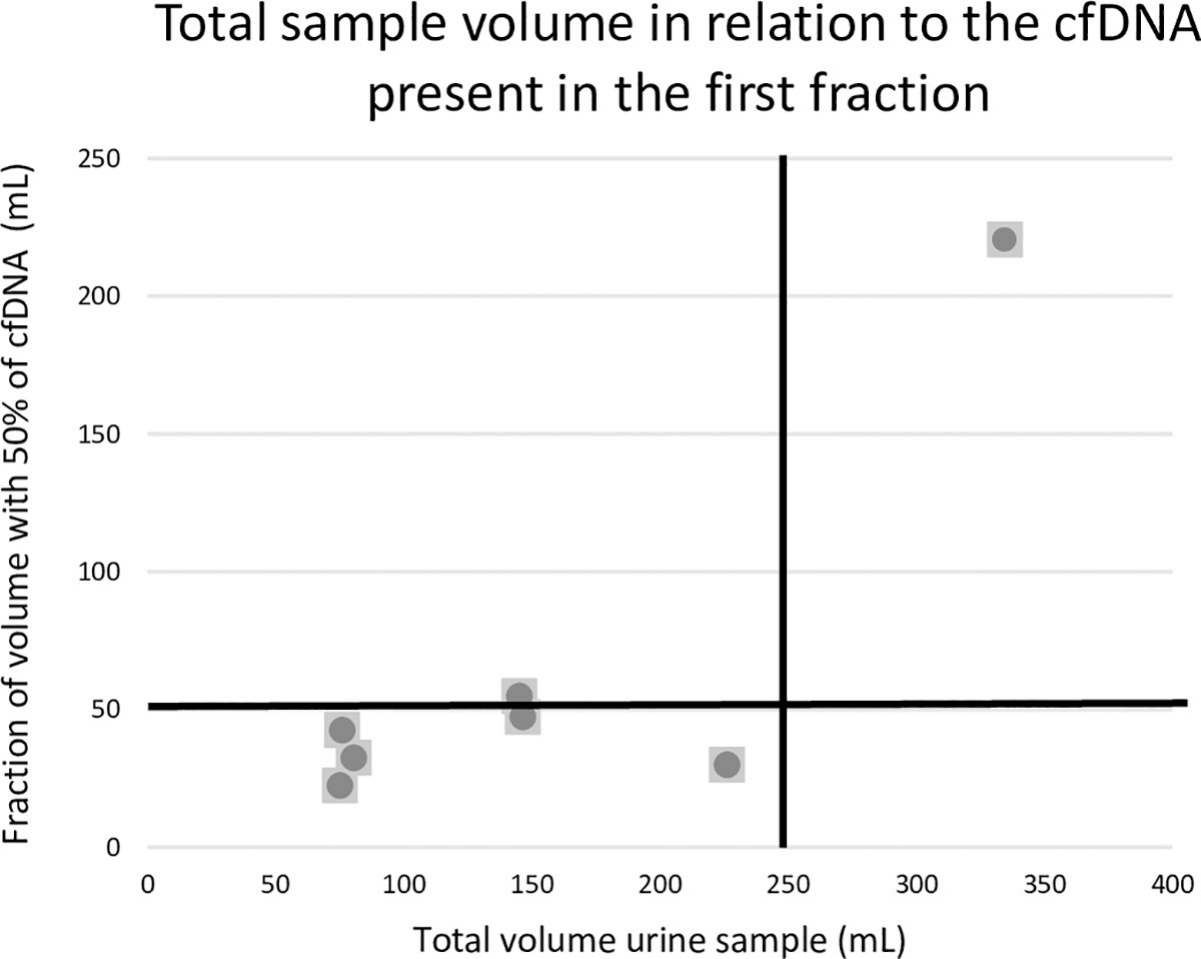
First-void versus random urine collection. Analysis was performed using the FragmentAnalyzer on all included samples. Each dot represents the fraction of a urine sample (in mL) of one HV that is needed to detect 50% of the cfDNA present in this urine sample. In urine samples with a small volume (dots on the left of the vertical black line), the first 50 mL of the urine sample suffices to detect 50% of the present cfDNA in the sample (dots below horizontal black line). In the only urine sample with a large volume that was tested (dots on the right of the vertical black line), the first 50 mL of the urine sample does not suffice to detect 50% of the present cfDNA in the sample (dots above horizontal black line). cfDNA = circulating cell-free DNA, gDNA = genomic DNA, HV = healthy volunteer.

The first experiment focused on the variation of cfDNA and gDNA content released within one urine sample. We analyzed urine samples from HVs and cancer patients (n = 7, HV 1–5 and P1-2). To study the difference of cfDNA and gDNA content within one sample, each sample was collected in different containers, so that the different fractions could be studied. The samples had a total volume ranging from 75 to 334.5 mL of urine. The Qubit Fluorometer was used to measure the concentration of total DNA. In one sample (HV1), the first 50 mL of urine only contained a minority (±28%) of the total DNA. This sample had the largest total volume. In three samples (HV3, 4 and 5) this first-void of 50 mL consisted of the majority (>80%) of total DNA. In the three remaining samples (HV2, P1 and P2), the first-void contained approximately half of the total DNA (Figs 2 and 3). These results were in agreement with ddPCR results. The samples from HV3, 4 and 5 showed a peak of total DNA in the first-void whereas the other samples (HV1, HV2, P1, P2) show a peak in (a fraction of) both the first-void and the remaining void (Fig 2). Samples were also studied with the FragmentAnalyzer to obtain information about the length of the fragments. Similar to the total DNA concentrations measured with Qubit and ddPCR, only a minority of the cfDNA was present in the first 50 mL of the total collection of urine (334.5 mL) from HV1. In one sample (HV3) the first-void contained the majority (>80%) of cfDNA. In five other samples (HV2, HV4, HV5, P1 and P2) the first-void urine (= 50 mL) contained approximately 50–60% of the cfDNA (Fig 2). P1 is known to have a *KRAS* mutation (c.35G>A p.Gly12Asp exon 2; MAF 33.0%), based on the formalin-fixed paraffin-embedded (FFPE) material. In contrast *KRAS* mutations could not be detected in the matched urine samples. Matched blood samples were not available. In conclusion, the first-void is not the only fraction of a urine sample that contains high amounts of (cf)DNA. Nevertheless, in samples with a relatively small volume (urine samples of HV2, HV3, HV4, HV5, P1 and P2) more than 50% of the cfDNA can be found in the first 50 mL of the urine sample (Fig 3).

### Collection time points

**Fig 4:**
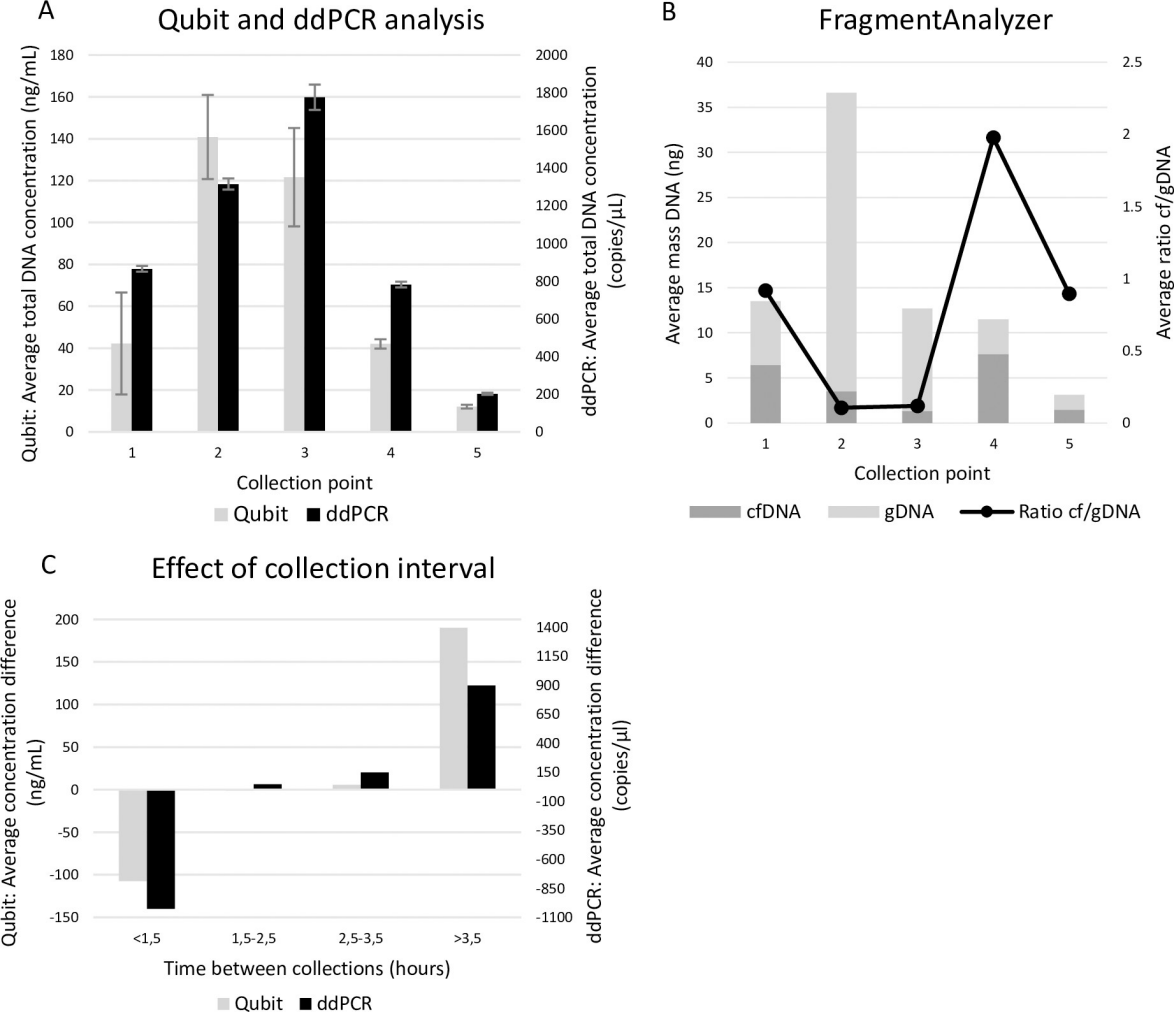
Collection time points. Different collection points were investigated throughout one entire day starting with the first morning urine with a maximum of five consecutive samples being collected. Results from four HVs are shown as an average for each collection point. A: Total DNA concentrations analyzed with Qubit fluorometer and ddPCR. B: Amount of cfDNA and gDNA as determined with the FragmentAnalyzer. C: The effect on time interval between two consecutive collection points on the total concentration measured with Qubit and ddPCR. cfDNA = circulating cell-free DNA, ddPCR = digital droplet PCR, gDNA = genomic DNA, HV = healthy volunteer.

In the second experiment, we focused on the identification of the most optimal time point for urine collection. Therefore, we studied the urine samples from HVs (n = 4) that were collected throughout the day starting with the first morning urine. Qubit and ddPCR measurements show that instead of morning urine, the second or third collection of the day yielded the highest concentration of total DNA (Fig 4A). When comparing the cfDNA and gDNA concentration using the FragmentAnalyzer, the first, fourth and fifth collection points had a relatively high ratio in comparison to the second and third collection point. However, their overall DNA concentrations were considerably lower compared to the other collection points (Fig 4B). To better understand these findings, we have investigated the effect of the collection interval on the amount of DNA in the samples. Our data showed that when a urine collection is followed by a second collection too close after the first, a significant drop in DNA concentration can be observed (Fig 4C). More specifically, a large drop in concentration was observed when the second sample was taken within less than 1.5 hours of the preceding collection. A collection interval of 1.5 to 3.5 hours did not result in any large differences. The total DNA concentration increased again when samples were collected more than 3.5 hours apart.

### Effect of preservatives

**Fig 5:**
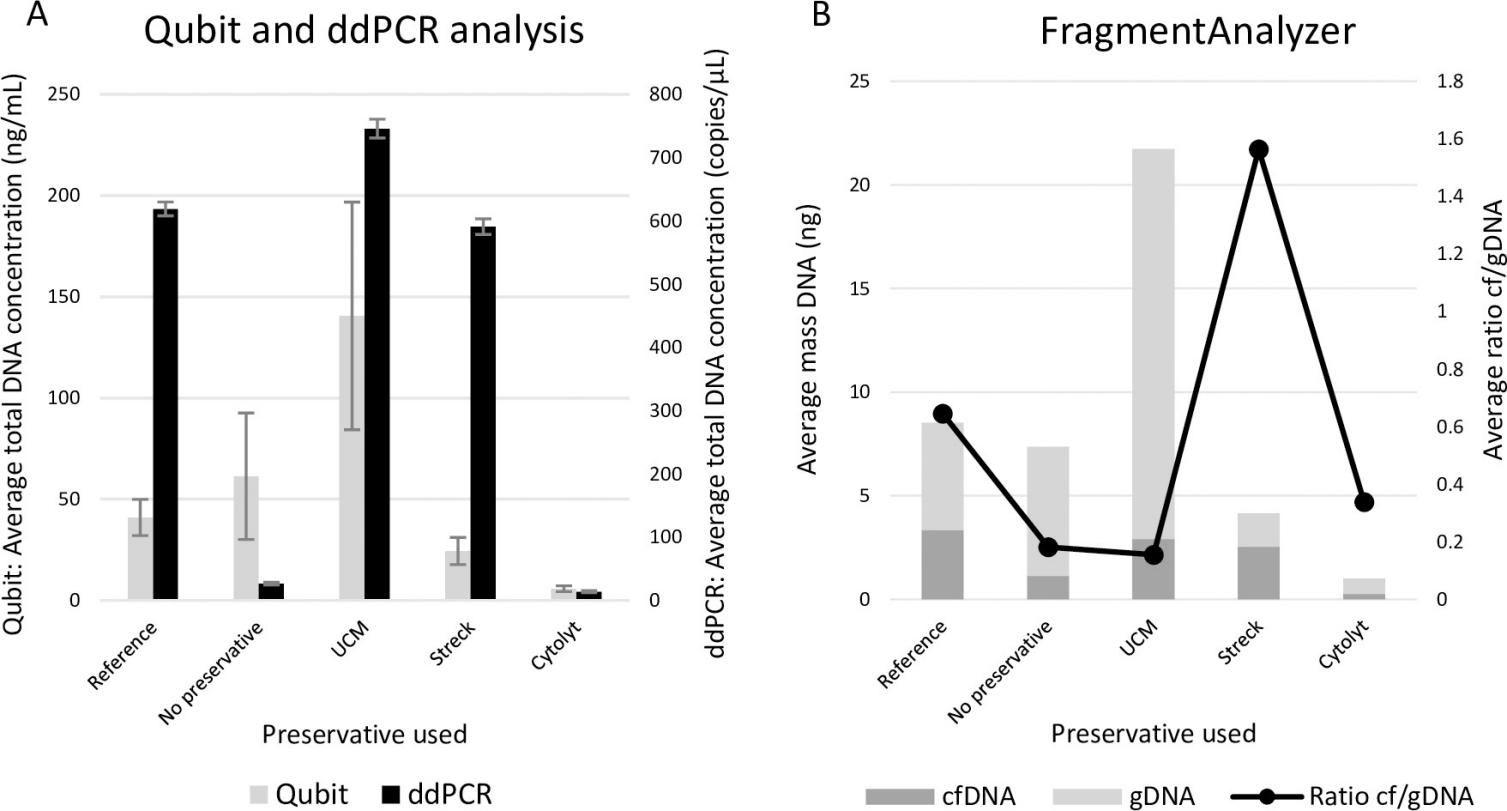
Effect of preservatives. The reference fractions were processed immediately after collection. The other fractions were stored for one week at RT with or without the addition of a preservative. Results from all included samples are shown as an average for each fraction. A: Total DNA concentrations analyzed with Qubit fluorometer and ddPCR. B: Amount of cfDNA and gDNA as determined with the FragmentAnalyzer. cfDNA = circulating cell-free DNA, ddPCR = digital droplet PCR, gDNA = genomic DNA, RT = room temperature, Streck = cfDNA Urine Preserve, UCM = Urine conservation medium.

Urine samples of HVs and cancer patients (n = 8) were divided in multiple fractions and stored with preservative for 1 week at RT to test the effect of three different preservatives (Streck, UCM and Cytolyt). Quantification of total DNA by Qubit and ddPCR did not result in similar results in all conditions (Fig 5A). However, it is clear that storing urine samples for one week at RT without any preservative results in a depletion of the majority of DNA suitable for PCR. Similar, the urine fraction stored with the Cytolyt preservative did not contain much DNA after one week. A good preservation of total DNA (suitable for ddPCR) was seen when urine was stored with UCM or Streck preservative (Fig 5A). In order to fully comprehend the capacity of the preservatives, we analyzed all fractions with the FragmentAnalyzer for the presence of cfDNA. The two conditions (without preservative and with Cytolyt) in which the preservation of DNA (suitable for PCR) failed (Fig 5A), both contained a slightly higher amount of gDNA compared to cfDNA resulting in a low cfDNA/gDNA ratio (Fig 5B). The two other preservatives (UCM and Streck) that yielded a high amount of total DNA (Fig 5A) highlight an important difference between them. The FragmentAnalyzer showed a high preservation of gDNA fragments within the UCM fractions, while a higher percentage of cfDNA was seen in the Streck preservative (Fig 5B). The FFPE tissue of the patients was tested using the ddPCR method. Tissue of one of the patients showed clear *PIK3CA* H1047R positivity (MAF 6.4%). Due to the limited volume of each fraction, all the urine samples were *PIK3CA* negative. Matched blood samples to study the mutational status were not available.

### Effect of storage temperature and time

**Fig 6:**
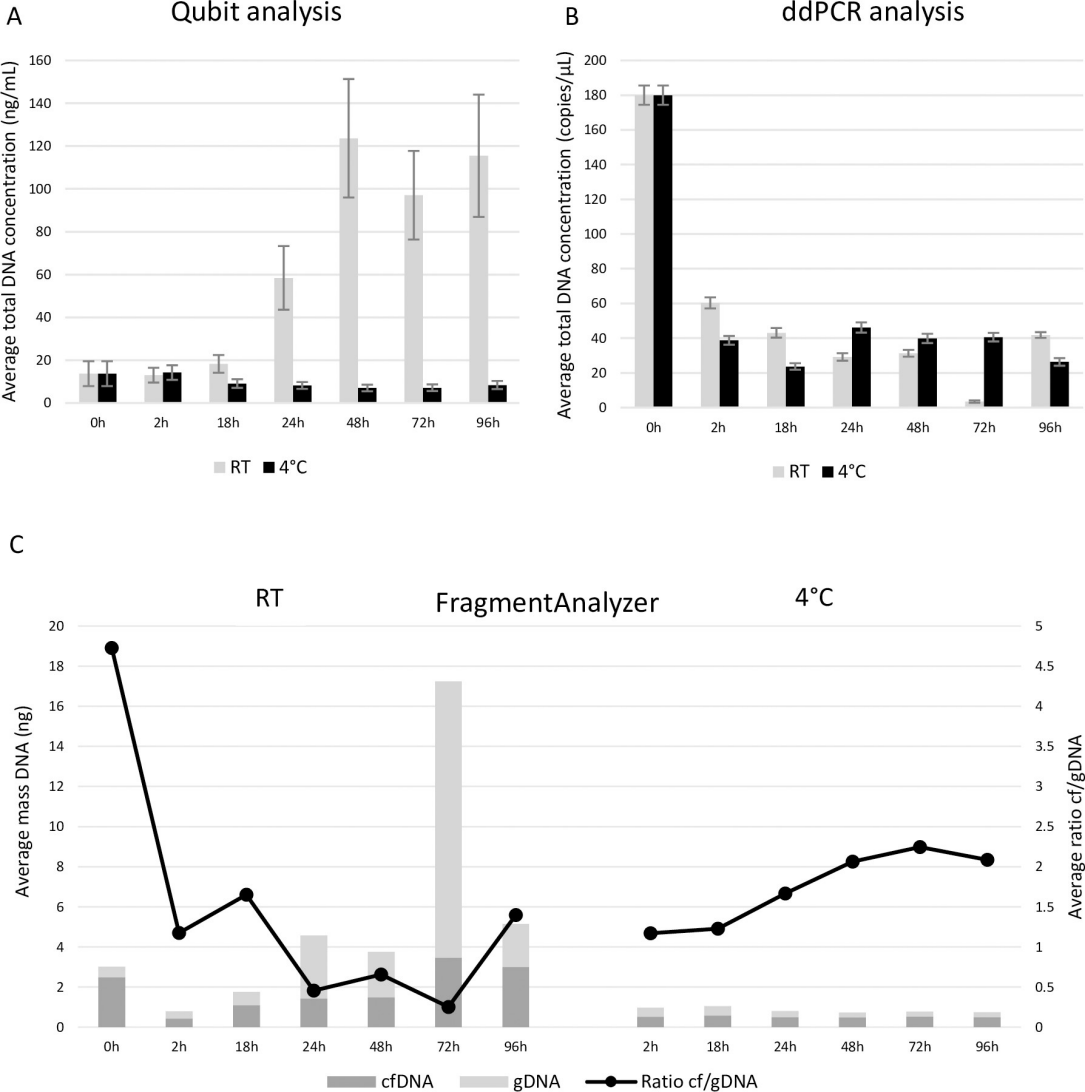
Effect of storage temperature and time in fresh urine samples. The reference fractions (0h) were processed immediately after collection. The other fractions were stored at RT or 4°C with a varying storage time. Results from all included samples are shown as an average for each fraction A: Total DNA concentrations analyzed with Qubit fluorometer. B: Total DNA concentrations analyzed with ddPCR. C: Amount of cfDNA and gDNA as determined with the FragmentAnalyzer. cfDNA = circulating cell-free DNA, ddPCR = digital droplet PCR, gDNA = genomic DNA, RT = room temperature.

Since (cf)DNA seems to be depleted after one week of storage at RT, we wanted to investigate both the dynamics of this DNA degradation and to what degree storage at 4°C could slow down this process. To this end, we stored fractions of urine without preservative (n = 6) between 0 and 96 hours at RT and at 4°C. Qubit and ddPCR data both showed that storage at 4°C results in more stable sample preservation up to 96 hours (Fig 6A and 6B). At RT, results showed more variety. Qubit analysis showed an increase in total DNA concentration after 24 hours of storage (Fig 6A), while ddPCR results showed more stable results with a slight decrease of DNA concentration over time (Fig 6B). The cfDNA/gDNA ratio measured with the FragmentAnalyzer also seemed more stable within the fractions stored at 4°C when compared to the fractions stored at RT (Fig 6C).

**Fig 7:**
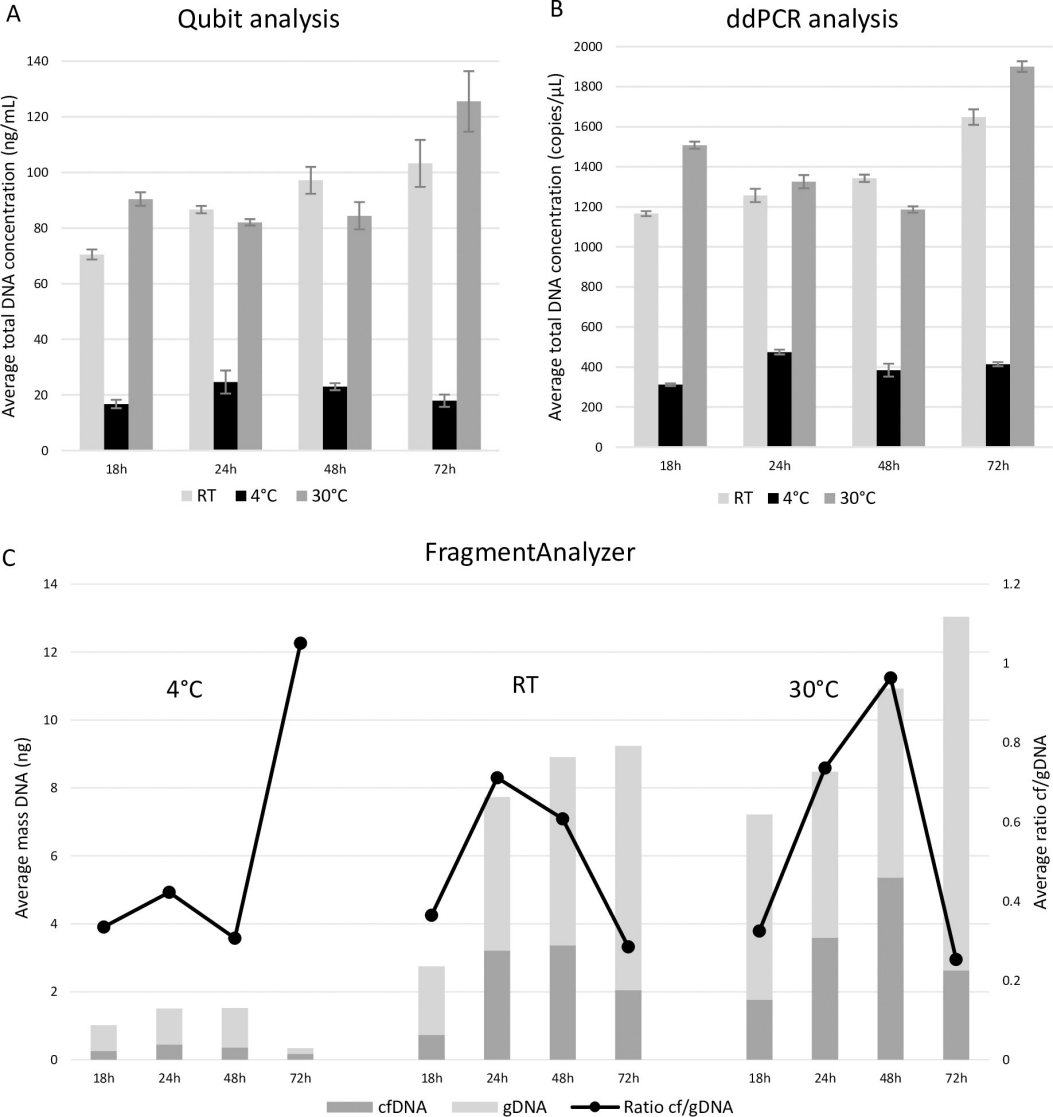
Effect of storage temperature and time in urine samples to which Streck preservative was added. The fractions were stored at 4°C, RT or 30°C for a varying storage time and supplemented with Streck preservative. Results from all included samples are shown as an average for each fraction. A: Total DNA concentrations analyzed with Qubit fluorometer. B: Total DNA concentrations analyzed with ddPCR. C: Amount of cfDNA and gDNA as determined with the FragmentAnalyzer. cfDNA = circulating cell-free DNA, ddPCR = digital droplet PCR, gDNA = genomic DNA, RT = room temperature, Streck = cfDNA Urine Preserve.

Matched FFPE tissue to show the presence of tumor biomarkers was not available. However, *KRAS G12/G13* variants were detected in the plasma of both patients (MAF 2.4 and 10.2%). In the urine samples of the first patient *KRAS* mutated cfDNA was detected in the reference fraction (MAF 0.05%) and in the fraction stored at 4°C for 72h (MAF 0.1%). As the urine volume was too small to prepare fractions at RT, we could not study *KRAS* mutated cfDNA on RT of this patient. In the second patient *KRAS* positivity was found in the urine fractions stored at RT for 24h, 48h and 96h (MAF 1.5%; 2.1% and 1.3% respectively) and in the fractions stored at 4°C for 24h, 48h and 96h (MAF 1%; 2.9% and 1% respectively). As our previously described experiments (Fig 5) clearly showed that transport with Streck preservative is preferred for cfDNA analysis, we also investigated different transport times and temperatures in urine samples with this preservative (n = 4). In this part, storage times varied between 0 and 72 hours, representing possible transit times of the samples up to three days. The investigated temperatures in this section were 4°C, RT and 30°C, mimicking transport conditions in Europe. Both Qubit and ddPCR analysis showed an overall lower DNA concentration for the fractions supplemented with Streck stored at 4°C (Fig 7A and 7B). The Streck fractions stored at RT and 30°C showed a higher stability over time. When looking at fragment analysis results, the fractions stored at 4°C had the lowest overall DNA content confirming the loss of DNA seen with Qubit and ddPCR analysis (Fig 7C). Fragment analysis results also showed that both cfDNA and gDNA content had diminished when fractions were stored at 4°C. Although the fractions stored at RT and 30°C had a higher DNA content, the cfDNA/gDNA ratio was comparable to the ratio seen in the fractions stored at 4°C (Fig 7C).

### Preparation of cell-free urine by centrifugation

**Fig 8:**
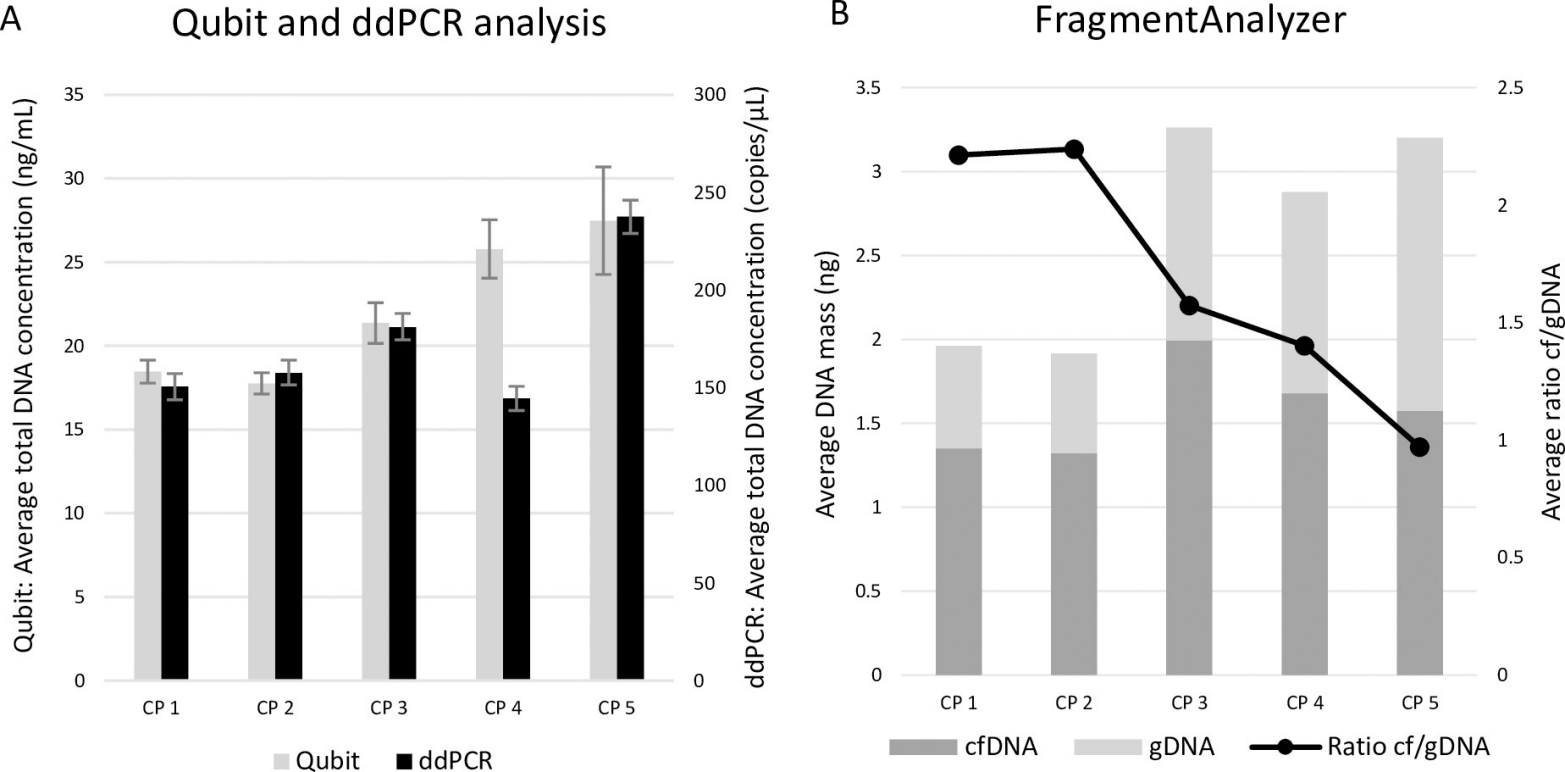
Preparation of cell-free urine by centrifugation. Five different centrifugation protocols were evaluated using fresh urine samples of HVs. A: Total DNA concentrations analyzed with Qubit fluorometer and ddPCR. B: Amount of cfDNA and gDNA determined with the FragmentAnalyzer. cfDNA = circulating cell-free DNA, CP = centrifugation protocol, CP 1 = 750g 10min + 2,680g 10min, CP 2 = 750g 10min + 3,350g 15min, CP 3 = 750g 20min + 2,680g 10min, CP 4 = 750g 20min, CP 5 = 500g 10min, ddPCR: digital droplet PCR, gDNA = genomic DNA, HV = healthy volunteer.

We aimed to identify a centrifugation protocol for urine samples that efficiently removes the cellular components without any loss of cfDNA. For fresh urine (without preservative) from HVs (n = 3) five centrifugation protocols were tested with varying centrifugation speed and duration (Table 5 top panel). Based on Qubit measurements, in 2/3 of the HVs, the one-step protocol with the lowest centrifugation speed (CP5: 500g 30min) generated higher yields of total DNA in comparison to the other protocols (CP1: 750g 10min+2,680g 10min; CP2: 750g 10min+3,350g 15min; CP3: 750g 20min+2,680g 10min and CP4: 750g 20min) (Fig 8A). Results generated with ddPCR were similar. Fragment analysis on the other hand demonstrated that the amount of cfDNA obtained with each protocol is quite similar and that the amount of gDNA contamination strongly varied between the protocols. Two two-step protocols (CP1: 750g 10min+2,680g 10min; CP2: 750g 10min+3,350g 15min) generated less gDNA compared to the three other protocols (Fig 8B).

**Fig 9:**
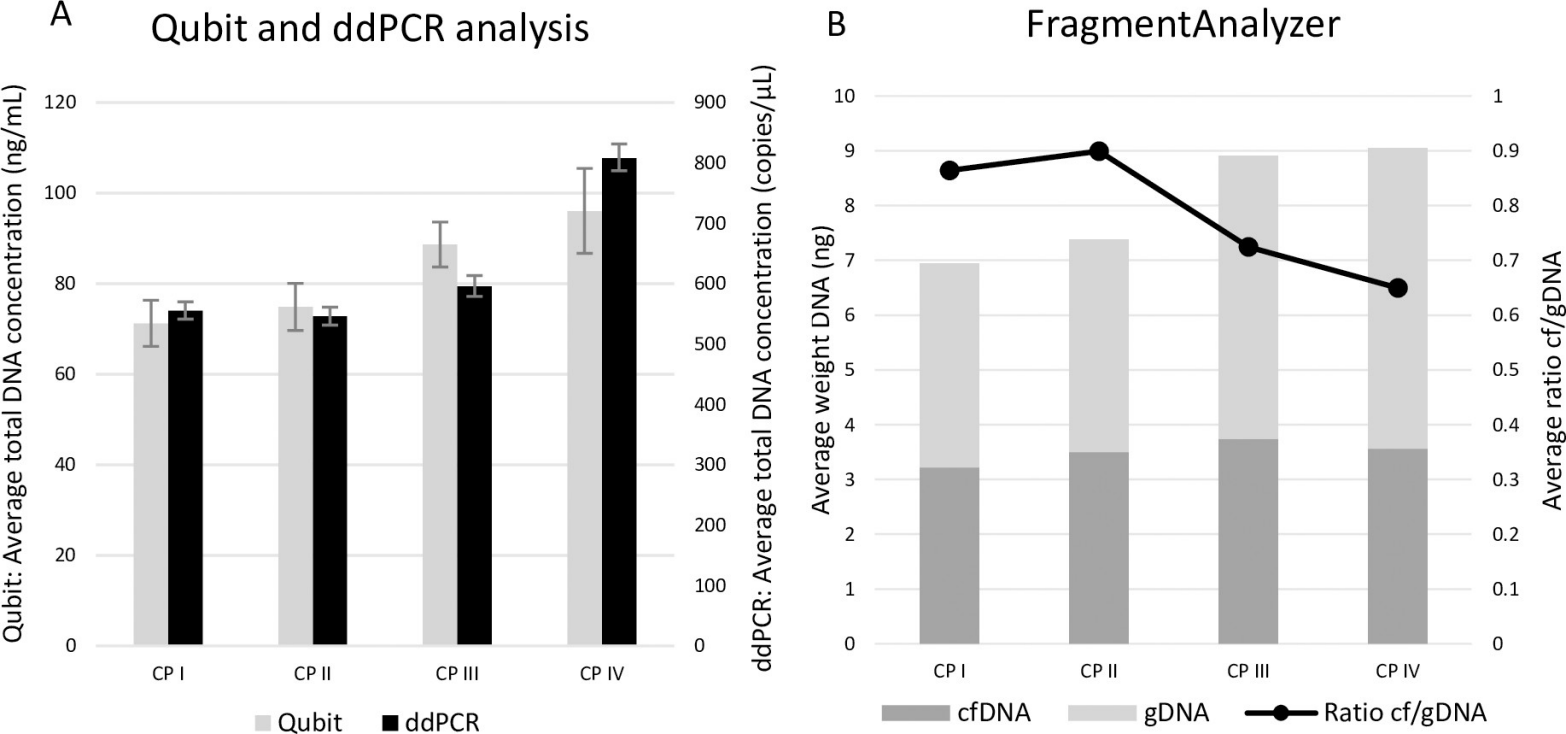
Evaluation of cfDNA and gDNA concentration for the investigation of centrifugation protocols with preservatives. Four different centrifugation protocols were evaluated using urine samples with preservative of cancer patients and HVs. A: Total DNA concentrations analyzed with Qubit fluorometer and ddPCR. B: Amount of cfDNA and gDNA determined with the FragmentAnalyzer. cfDNA = circulating cell-free DNA, CP I = 4,000g 10min, CP II = 3,000g 15min, CP III = 2,680g 10min, CP IV = 750g 10min, ddPCR = digital droplet PCR, gDNA = genomic DNA, HV = healthy volunteer.

To identify the most optimal centrifugation protocol for urine samples with preservative (cfDNA Urine Preserve, Streck), we have also tested four different centrifugation protocols (Table 5 bottom panel) on samples from HVs (n = 4) and cancer patients (n = 4) supplemented with preservative. In the majority (6/8) of samples, Qubit and ddPCR analysis showed that the low-speed centrifugation protocol 750g 10min (CP IV) generated a higher yield of total DNA (Fig 9A). FragmentAnalyzer results clearly showed that the higher the centrifugation speed, the lower the amount of gDNA contamination in the sample. The cfDNA content was highly similar when comparing the different centrifugation protocols. When taking into consideration both the amount of cfDNA and the purity of cfDNA (= cfDNA/gDNA ratio), protocol CP I or II (CPI: 4,000g 10min; CPII: 3,000g 15min) might be the most ideal centrifugation protocol for urine collected in preservative. In both HVs and patients, urine samples that were centrifuged at 750g for 10min (CP IV) resulted in the highest percentage of gDNA contamination (Fig 9B). Based on FFPE testing using ddPCR, one of the four included patients possesses a *PIK3CA* E545K mutation (MAF 6.1%). Three out of four urine fractions (fractions centrifuged at CPI: 4,000g 10min; CPII: 3,000g 15min and CPIV: 750g 10min) of this patient also showed positivity for this mutation (MAF 0.03% in each fraction). Unfortunately, matched blood samples were not available.

## Discussion

In this study, we have investigated several pre-analytical conditions in order to establish some good practice guidelines on the use of urine samples for cfDNA analysis in the management of cancer patients. Vorsters *et al*. showed that the first-void urine (50 mL) contained a higher concentration of viral DNA than the remaining void [32, 33]. We saw similar results in six of the seven samples. Six of these urine samples were highly concentrated as the total urine volume ranged from 75–226 mL. The sample with the higher volume did not contain the majority of the cfDNA in the first void (Figs 2 and 3). These data are also in agreement with the observation of Reckamp *et al*. showing an increased sensitivity for urinary *EGFR* cfDNA detection in NSCLC patients when at least 90–100 mL of urine could be obtained for analysis [34]. As most commercial cfDNA isolation kits cannot process such large volumes, the need for concentrating urine samples prior to isolation is becoming apparent. Furthermore, we observed that the concentration cfDNA in urine samples of women is higher than in the concentration of cfDNA in urine samples of men. This can be explained due to the fact that urine samples of women not only contain transrenaly cleared cfDNA but also genital cfDNA, which originates from genital secretions (contain biomarkers form cervix, vagina…) that accumulates in the small labia.

For cancer patients with known *KRAS* or *PIK3CA* mutations (as determined by tissue testing) we also compared the presence of oncogenic mutations in plasma and urine samples. In all cases the concentration of mutated cfDNA was higher in plasma than in urine. This observation may have a biological origin (glomerular filtration, kinetics of cfDNA in urine) and/or could be due to technical aspects (sample preservation/ efficiency of DNA isolation from large volumes). Our initial hypothesis that the first urine collection of the day contains the highest concentration of total DNA was incorrect in the majority of cases [35]. (Fig 4A). The different urine collections from a single person during the day showed a high variability in cfDNA and gDNA content. Our data show that consecutive samples should be separated with at least 1.5 hours in between samples to ensure sufficient cfDNA concentration (Fig 4C). We hypothesize this observation might be explained by the glomerular filtration rate limiting the amount of cfDNA that can be filtered from the bloodstream when the time interval between two urinations is too short [36]. Uptake of fluids was not recoded in this study and could give further insights into how a urine sample with a high cfDNA concentration can be obtained.

Another important pre-analytical factor, storage and transport conditions of urine samples, was highlighted in this study. Even when samples are processed immediately, the use of a suitable preservative is recommended to avoid cell lysis and DNA degradation (Fig 5A). In samples supplemented with Cytolyt a low total DNA concentration with relative high gDNA content was observed. (Fig 5A and 5B). Cytolyt is designed to preserve cells instead of cfDNA offering an explanation for our findings [37]. UCM and Streck preservative on the other hand both yielded a high total DNA concentration (suitable for ddPCR) (Fig 5A), while only Streck also preserved a high proportion of cfDNA (Fig 5B). These findings indicated the need for a preservative that is designed specifically for the purpose of preserving cfDNA in urine samples. Some of these findings were also observed by a different research group [31], like the necessity of using a preservative when storing urine at RT. In addition, it might also be important to supplement urine samples with an anti-fungal and anti-bacterial compound, which was not included in our study, avoiding the growth of non-human DNA [31]. This potential growth of fungal or bacterial DNA in our samples could explain the variations seen between Qubit and ddPCR measurements. The presence of fungal or bacterial DNA will results in an increase in gDNA contamination.

Our data suggested a preservative should always be used (Fig 5). Storing fresh urine samples at RT resulted in samples with a variable yield of cfDNA and a high level of gDNA contamination in the majority of cases. However, storing urine samples at 4°C results in samples with a low total DNA concentration and a relative good cf/gDNA ratio. Armstrong *et al*. studied the preservation of miRNAs in urine, which showed a better miRNA recovery after storage at 20°C when compared to 2°C, indicating an important difference between DNA and RNA in urine [30]. This maybe explained to the fact that more tumor cells will lyse after 72h or 96h than after 24h, which secrete cf tumor DNA. However, when a preservative can be used (e.g. for the transportation of urine samples collected at home to the hospital), samples should not be transported at 4°C. The temperature of 4°C was found to be incompatible with the Streck preservative, resulting in a rapid decrease of total DNA (both cfDNA and gDNA fractions) within 18 hours after collection. These observations are in agreement with the specifications on the datasheet of Streck. Instead, cfDNA (and gDNA) concentrations during transportation of the samples up until 72 hours were found to be stable when transported at RT or even higher temperatures (Fig 7).

For qualitative analysis of cfDNA it is also important to use a suitable centrifugation protocol to avoid contamination by gDNA derived from cells in the urine. To remove these cells from the sample prior to cfDNA isolation when studying fresh urine samples, a short, slow-speed step is necessary (Fig 8). Unfortunately, these slow-speed protocols do not clear all the cells in the sample, causing cell lysis during thawing or during cfDNA isolation. To acquire the best results, a second short but high-speed step should be added to the protocol. When studying urine samples with preservative, one-step short low-speed centrifugation protocols resulted in the highest total DNA concentrations (Fig 9A). Unfortunately, FragmentAnalyzer results indicate all centrifugation protocols still yield a high amount of gDNA in all tested samples (Fig 9B). Although differences are not significant, our results suggest to use the centrifugation protocol 750g 10min+2,680g 10min for fresh urine samples and 4,000g 10min or 3,000g 15min when Streck preservative is added to the sample.

In this study all samples were extracted using the Quick DNA Urine Kit from ZymoResearch as this kit allows a sample volume of 40 mL. However, this kit is more appropriate for isolation of DNA fragments >100bp. As a fraction of the transrenal cfDNA (smaller than 100bp [17, 18]) might be missed, it is of paramount importance that a thorough investigation of several cfDNA extraction methods from urine should be performed. Oreskovic *et al*. has investigated the analytical performance of several cfDNA extraction methods, focusing on short cfDNA fragments from urine [38]. Their data indicate that a hybridization capture based method is the most suited cfDNA extraction method for short DNA fragments. The purpose of this study was to examine different pre-analytical conditions to create an optimal workflow for urine sample processing. Since the number of HVs and patients included in the study was limited, the trends we have observed in our study should be investigated in a larger study population to confirm our findings. Finally, it is important to note that even though the volume of each urine sample that was studied was relatively small, we succeeded in detecting *KRAS* or *PIK3CA* variants in some samples. Since the detected concentration of tumor biomarker cfDNA was around the limit of detection, no conclusion concerning the superiority of one of the conditions could be made based on this data.

Based on our pilot study some suggestions about the pre-analytical conditions of urine samples can be made. We would recommend to isolate the whole urine sample when studying molecular alterations in the cfDNA. In addition, the collection interval between two urine collections of urine must be at least 1.5 hours, to prevent a large drop in total DNA concentration. Fresh urine should be processed as soon as possible after the sample was collected. When taking into consideration both the amount of cfDNA and the purity of cfDNA in fresh urine samples 750g 10min+2,680g 10min might be the most ideal centrifugation protocol. If it is not possible to process the urine sample immediately after collection, preservative (cfDNA Urine Preservative, Streck) should be added. Samples collected in this preservative should preferentiality be stored at RT and centrifuged at 4,000g 10min or 3,000g 15min based on our results.

## Abbreviations

bp: Base pairs
cfDNA: Circulating cell-free DNA
cfRNA: Circulating cell-free RNA
CP: Centrifugation protocol
CRC: Colorectal cancer
CTCs: Circulating tumor cells
CV: Coefficient of variation
ddPCR: digital droplet PCR
EGFR: Epidermal Growth Factor Receptor
EVs: Extracellular vesicles
FDA: Food and drug administration
FFPE: Formalin-fixed paraffin-embedded
gDNA: Genomic DNA
HPV: Human Papillomavirus
HV: Healthy volunteer
MAF: Mutant allele frequency
miRNA: MicroRNA
NSCLC: PDAC: Non-small cell lung cancer Pancreatic ductal adenocarcinoma
RT: Room temperature
TEPs: Tumor educated platelets
UCM: Urine conservation medium
UZA: University hospital of Antwerp
